# Dual-balloon overlapping post-dilation for stent optimization in markedly dilated coronary artery

**DOI:** 10.3389/fcvm.2026.1818706

**Published:** 2026-04-29

**Authors:** Yan Wang, Mingjing Shao, Jie Zhang, Tianshu Xie, Peng Yang, Qian Wang, Tong Gao, Jiejia Liang, Qian Li, Weidong Hong, Juan Zhang, Feng Gao, Hongfei Yang, Lisha Hao, Xiaofang Yu, Ke Dong, Changhong Zhao, Di Wang, Xianlun Li, Jiangquan Liao

**Affiliations:** 1 Department of Integrative Medicine Cardiology, China-Japan Friendship Hospital, Beijing, China; 2 Graduate School, Beijing University of Chinese Medicine, Beijing, China; 3 Department of Cardiology, Tumoteyouqi Hospital, Baotou, China; 4 Cardiovascular Department, Henan Provincial Hospital of Traditional Chinese Medicine, Zhenghzou, China

**Keywords:** coronary aneurysm, dual-balloon technique, intravascular imaging, post-dilation, stent malapposition

## Abstract

This manuscript describes the successful use of a dual-balloon overlapping post-dilation technique to achieve optimal stent expansion and apposition in a markedly dilated right coronary artery (RCA) where conventional high-pressure post-dilation with a single, maximum-sized non-compliant balloon failed.

## Case presentation

A 53-year-old male with a history of an old myocardial infarction ten years prior, managed with thrombus aspiration and anticoagulation without stenting due to aneurysmal dilation, presented with an acute inferior ST-segment elevation myocardial infarction (STEMI). After successful emergency thrombolysis, subsequent coronary angiography revealed a severe stenosis in the proximal RCA, with significant aneurysmal dilation distally. The patient was referred to our center for elective percutaneous coronary intervention (PCI).

## Intervention

**Figure 1:**
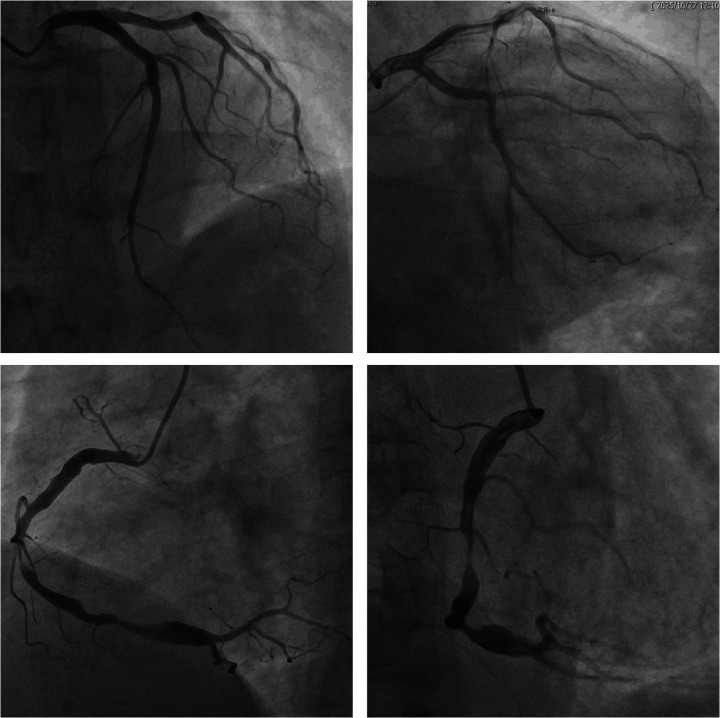
Coronary angiography image. The angiogram shows severe stenosis in the mid and distal segments of the right coronary artery, followed by marked post-stenotic aneurysmal dilatation of the distal vessel.

Coronary angiography was performed, and severe stenosis and aneurysmal dilatation was found in RCA (Figure 1). Planned balloon pre-dilation of the RCA was performed. Intravascular ultrasound (IVUS) confirmed aneurysmal dilation adjacent to the lesion, with a maximum vessel diameter of 7.43 × 6.35 mm (Supplementary Video S1). A 4.0-mm drug-eluting stent (DES), which was the largest diameter available at our center, was deployed, intended for the proximal slope of the aneurysm. However, patient movement during deployment resulted in the distal portion of the stent landing over the widest segment of the dilation. Despite repeated high-pressure post-dilation with a 5.0-mm non-compliant balloon (the maximum available), IVUS demonstrated persistent stent malapposition (Supplementary Video S2).

**Figure 2:**
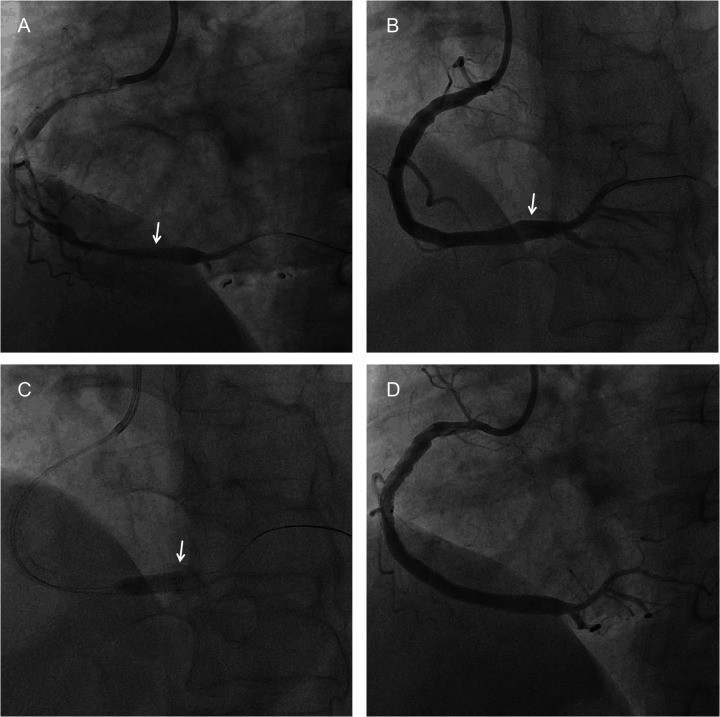
PCI procedure. **(A)** The initial intended positioning of the distal stent. **(B)** The stent position after deployment. **(C)** Balloon dilation using the DBOPD technique. **(D)** The final result after balloon dilation.

**Figure 3:**
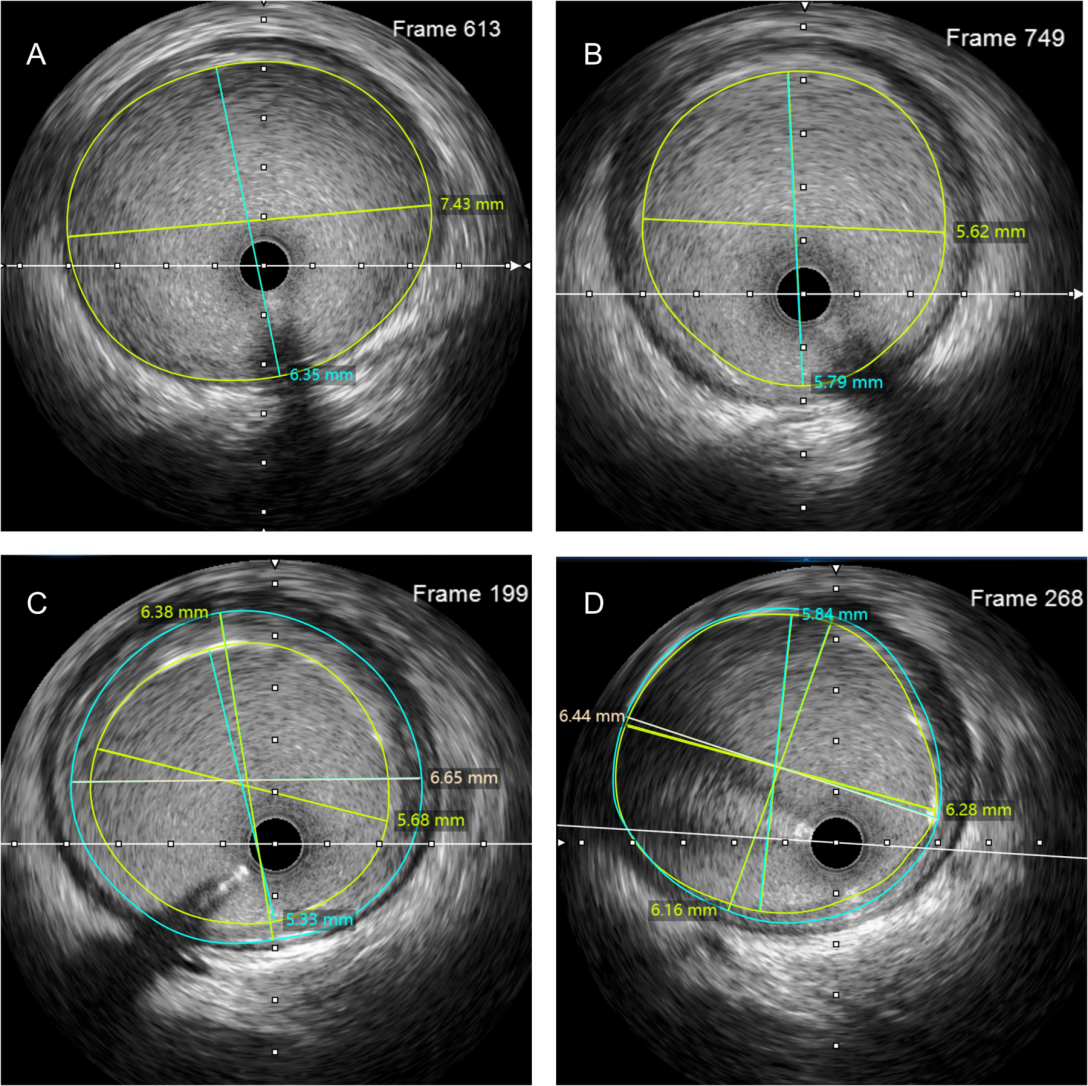
IVUS image during PCI. **(A)** Maximum diameter of the distal aneurysmal dilatation. **(B)** Initial intended positioning of the distal stent. **(C)** Stent expansion morphology after initial post-dilatation. **(D)** Final result after DBOPD.

To mitigate the risk of acute or subacute stent thrombosis, a dual-balloon overlapping post-dilation (DBOPD) technique was employed. This technique, conceptually derived from the buddy balloon principle applied in a post-dilation setting, involved the simultaneous advancement of two 5.0-mm non-compliant balloons of unequal length through a 7-French guiding catheter. The balloons were positioned with their distal markers aligned at the stent's distal edge, creating a staggered proximal marker profile. Simultaneous inflation was performed at 8 atm, then sequentially increased to 10 atm, 12 atm, with IVUS assessment after each inflation step to confirm progressive apposition and exclude vessel injury. After achieving satisfactory expansion at the distal segment, the shorter balloon was withdrawn, and the other was pulled back to the stent's mid-portion and inflated again to optimize the expansion gradient (Figures 2, 3). Final IVUS confirmed excellent stent apposition and expansion without edge dissection or vessel injury (Supplementary Video S3).

Quantitative IVUS analysis after single-balloon post-dilation revealed: MSA at the malapposed segment of 4.8 mm^2^ (vs. distal reference area of 6.2 mm^2^; 77% of reference), maximal axial stent-to-wall distance of 0.9 mm, and longitudinal malapposition length of 8.5 mm. These findings met established criteria for both significant underexpansion (MSA <5.5 mm^2^ and <90% of distal reference) and major malapposition (distance ≥0.4 mm, length >1 mm) (1). After DBOPD, final MSA improved to 7.6 mm^2^ with complete strut apposition.

**Figure 4:**
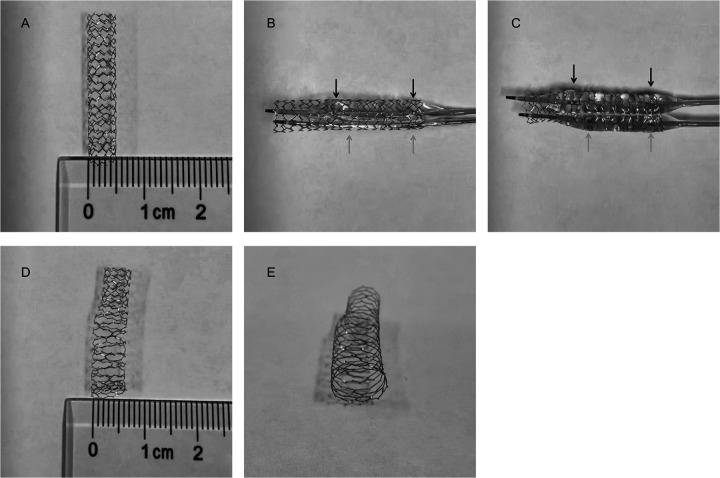
DBOPD technique *ex vivo* demonstration. **(A)** 4.0 × 24 mm DES expanded with a 5.0 × 15 mm post-dilation balloon (12atm, NOMINAL pressure). **(B)** Two balloons positioning for the DPOPD technique. **(C)** Simultaneous inflation of balloons. **(D)** Stent configuration after full expansion with the DPOPD technique (12atm, NOMINAL pressure). E: Cross-sectional stent morphology after DPOPD expansion (IVUS confirms near-circular rather than oval lumen within the coronary artery).

An *ex vivo* demonstration of DBOPD technique was performed afterwards and shown in Figure 4.

## Discussion

Achieving adequate stent expansion and apposition in ectatic or aneurysmal coronary segments poses a significant challenge in interventional cardiology (2, 3). Stent placement must ensure complete lesion coverage while also meeting IVUS optimization criteria (e.g., minimum stent area ≥90% of the distal reference lumen area, or ≥5.5 mm^2^) (4, 5). Recent evidence from the RENOVATE-COMPLEX-PCI trial suggests that the combination of underexpansion and major malapposition portends a particularly high risk of adverse events (1). When the vessel diameter exceeds the maximum achievable expansion diameter of a single post-dilation balloon, conventional techniques fail. In our case, the malapposed segment met criteria for both pathologies, justifying the need for aggressive salvage intervention.

The DBOPD technique described here successfully overcame this limitation. Key procedural aspects include: (1) using a large-lumen guiding catheter (7-French or larger); (2) utilizing new balloons for optimal deliverability; (3) performing adequate single-balloon pre-dilation to facilitate the passage of two balloons; (4) selecting balloons of different lengths; and (5) employing a stepwise pressure increase under IVUS guidance to avoid vessel rupture. The effective expansion diameter achievable with this technique is estimated to be approximately 70% of the sum of the two balloon diameters, analogous to geometric principles in bifurcation kissing-balloon inflation (6, 7). The final sequential inflation creates a conical frustum shape, ensuring a smooth transition.

This technique holds potential for other scenarios involving stent malapposition due to large vessel caliber, such as in the left main coronary artery or when stents extend into the aortic ostium. Intravascular imaging is mandatory for guiding and validating the procedure. Intravascular imaging is mandatory for guiding and validating the procedure.

Coronary perforation is a recognized risk of high-pressure post-dilation, particularly when balloons are oversized relative to the vessel. Risk factors include balloon-to-artery ratio >1.3 and use of very high inflation pressures. To mitigate this risk in DBOPD, we recommend: (1) exclusive use of non-compliant balloons for predictable expansion; (2) stepwise pressure increase under continuous IVUS monitoring; (3) confirmation of intrastent balloon positioning before each inflation; and (4) immediate reassessment after each step to detect early warning signs of vessel overstretch.

Another important consideration when applying the DBOPD technique is the impact of overexpansion on stent integrity. Overstretching a drug-eluting stent beyond its labeled maximal diameter can reduce radial strength, potentially leading to stent fracture, geometric distortion, or altered drug elution profiles (8, 9). Bench testing has demonstrated that significant overexpansion can result in strut fracture and loss of scaffolding capacity, particularly in stent designs with closed-cell geometry (10). In our case, the 4.0-mm stent was expanded to accommodate a 7.43-mm vessel—an approximately 60% overexpansion relative to nominal diameter. While IVUS confirmed excellent acute apposition without visible strut malformation, the long-term radial strength of the overexpanded stent remains unknown. Therefore, we emphasize that DBOPD should be considered a salvage technique for carefully selected cases where the benefits of achieving apposition outweigh the potential risks of compromised stent integrity. Whenever feasible, selecting a stent with a larger nominal diameter or using dedicated large-vessel devices is preferable. Close clinical and imaging follow-up is mandatory in such cases.

We acknowledge a prior report by Kaya et al. (11) describing simultaneous double balloon angioplasty using two compliant balloons for post-dilation in a patient with right coronary artery ectasia. While both techniques employ two balloons, our DBOPD approach differs fundamentally. Non-compliant balloons were used to achieve predictable, controlled expansion; sequential protocol with specific balloons in length and size in the guidance of IVUS was utilized and summarized. To our knowledge, this is the first systematic description of DBOPD as a standardized salvage technique for managing severe underexpansion/malapposition in large coronary vessels.

## Conclusion

The dual-balloon overlapping post-dilation technique is a feasible and effective innovative approach to achieve optimal stent results in coronary arteries with diameters exceeding the limits of conventional single-balloon post-dilation. It represents a valuable solution for a challenging clinical problem.

## Data Availability

The original contributions presented in the study are included in the article/Supplementary Material, further inquiries can be directed to the corresponding author.

## Supplementary material

Supplementary Video S1 Post-dilation IVUS assessment

Supplementary Video S2 Single-balloon post-dilatation after stent implantation

Supplementary Video S3 Post DBOPD IVUS assessment
